# Formation and transfer patterns of key aroma compounds in cigar tobacco based on SBSE-GC-O-MS, pyrolysis-GC-O-MS and flue gas analysis correlation studies

**DOI:** 10.3389/fchem.2026.1755255

**Published:** 2026-02-17

**Authors:** Xue Wu, Xinhua Song, Tianze Liu, Xu Wang, Chao Wang, Wentao Zhao, Yuqing Dou

**Affiliations:** 1 Tobacco Research Institute, Chinese Academy of Agricultural Sciences, Qingdao, China; 2 Graduate School of Chinese Academy of Agricultural Sciences, Beijing, China; 3 Shandong China Tobacco Industry Limited Company, Jinan, China; 4 Shanghai New Tobacco Product Research Institute Co., Ltd., Shanghai, China

**Keywords:** flue gas, odor detection, pyrolysis, SBSE, sensory evaluation

## Abstract

The aromatic quality of cigars represents their core value, arising from the thermal transformation and migration of inherent components within tobacco leaves—a highly complex dynamic process. To systematically trace the evolution of key aroma compounds from tobacco leaves to smoke, this study pioneered a combined strategy of Stirred Barrel Sorption Extraction (SBSE), Pyrolysis (PY), and mainstream smoke capture. Utilizing Gas Chromatography-Olfactometry-Mass Spectrometry (GC-O-MS) coupled with Odor Activity Values (OAV) and correlation network analysis, a comprehensive analysis was conducted. Results revealed: The intrinsic aroma of cigar tobacco leaves is dominated by terpenes and carotenoid degradation products, presenting a “green and citrus fruit” profile. Pyrolysis fundamentally transforms the aroma spectrum into a “toasty sweetness and roasted nutty” profile centered on furan and pyrazine compounds. While smoke exhibited a balanced profile blending sweet, green, and fruity notes. Quantitative DTE revealed highly efficient nicotine transfer (30%), pyrazine pyrolysis enrichment (40%–50%), and phenol thermal generation (Thermal Generation Factor >8), with only 44% of Neophytadiene entering the smoke. Correlation analysis revealed that Phenol and 2-methoxy- in pyrolysis products positively correlated with multiple terpenes, aldehydes, and nitrogen-containing heterocyclic compounds in tobacco leaves, while Phenol showed negative correlations. The roasted aroma marker furfural in smoke significantly correlated with neophytadiene, a carotenoid degradation product in tobacco leaves. While the characteristic nutty aroma component 2,6-dimethylpyrazine showed a strong positive correlation with α-terpineol acetate in the tobacco leaf itself. This study elucidates the formation mechanism of cigar characteristic aromas at the molecular level, providing important theoretical basis for precise quality evaluation, scientific formulation design, and process optimization of cigar tobacco.

## 1 Introduction

With the integration of the global economy and rising consumption levels, the cigar market has experienced sustained growth, becoming an indispensable part of the global consumer goods market (Yu et al., 2023). As a high-perceived-value commodity, the sensory qualities of cigars are key determinants of their market value. Among the various sensory dimensions, aroma constitutes the core element in the perception of their quality. The aroma of cigar smoke originates from a complex array of components including aldehydes, ketones, esters, and alcohols. These elements not only construct the cigar’s unique flavor profile and sensory layers but also profoundly influence consumer preferences and purchasing decisions (Chen and Chen, 2022; Yan et al., 2022). According to consumer feedback, domestically produced cigars (such as CTL types) still exhibit significant shortcomings in aroma performance, primarily manifested in insufficient aroma intensity and monotonous flavor profiles (Cai et al., 2019; Chen et al., 2019). In contrast, cigars with rich aromatic characteristics—such as bean and nutty notes—are more favored (Sun et al., 2020), further confirming the central role of aroma in the overall sensory experience of cigars.

With the advancement of molecular sensory science, sensory-driven flavor characterization methods have become increasingly rigorous. Gas chromatography-olfactometry (GC-O) combines instrumental analysis with human olfaction, enabling effective identification of key aroma compounds along with their intensity and characteristics. This approach addresses the limitation of traditional GC-MS, which can only analyze volatile components without determining their contribution to aroma (Yu et al., 2022). Building upon this foundation, GC-O-MS integrates the sensory recognition capabilities of GC-O with the qualitative and quantitative functions of GC-MS, emerging as a powerful tool for systematically analyzing complex flavor systems. It is now widely applied across multiple sectors including food, tobacco, and flavor and fragrance industries. In food science, Jiang et al. (2023) conducted GC-O-MS analysis on aged citrus peel (Chenpi) matured for 6 years, identifying 42 aromatic compounds. They observed stable intensity in spicy, woody, and herbal aromas, while citrus and fruity notes gradually diminished or vanished. In tobacco research, Wang et al. (2024) analyzed five-year-aged cigar tobacco leaves, detecting 75 aroma compounds whose extracts primarily exhibited floral and fruity notes. Dou et al. (2024) used pyrolysis-gas chromatography-mass spectrometry (PY-GC-MS) to confirm 21 tobacco leaf aroma components. Furthermore, Song and Liu (2018) analyzed the smoke components of 18 domestic and imported cigars via GC-MS, detecting a total of 440 volatile compounds. Overall, existing research has explored tobacco leaf aroma components, thermal cracking behavior, and smoke composition from various perspectives, accumulating extensive data. However, previous studies have largely been confined to identifying components in single matrices (e.g., analyzing only tobacco leaves or only smoke) or relying on subjective sensory descriptions for correlations. This critical gap hinders our deep understanding of the flavor formation mechanism across the entire cigar production chain and constrains the precise regulation of product flavor design. Therefore, constructing a holistic flavor correlation model spanning from raw materials to smoking quality will become a key research direction for enhancing the sensory quality of cigars in the future.

This study proposes and implements for the first time a “three-stage tracking” integrated strategy. This approach involves simultaneously obtaining the key aroma-active components and dominant aroma profiles of the cigar tobacco itself (SBSE), pyrolysis simulant (PY), and mainstream smoke. Utilizing odor contribution value (OAV) screening and multivariate correlation network analysis, this approach aims to objectively and data-drivenly reveal the dynamic transfer, generation, and transformation patterns of aroma compounds throughout the complete chain: “leaf → pyrolysis → smokiness.” This approach identifies directly transferred aroma compounds and pyrolytically generated aroma compounds, predicts potential formation pathways and precursors for key aroma substances, and offers new insights into the mechanisms underlying cigar aroma formation.

## 2 Materials and methods

### 2.1 Materials, reagents, and equipment

Fourteen representative cigar tobacco leaf samples were selected from seven major production regions. All samples were provided by the Shandong Provincial Tobacco Administration, ensuring their varietal characteristics, curing, and aging conditions met industrial production standards. Detailed sample information is presented in Table 1. Sample Preparation and Storage: Upon arrival at the laboratory, all samples were immediately equilibrated for 72 h in a constant temperature and humidity chamber (20 °C ± 2 °C, 60% ± 5% RH). Subsequently, leaves were destemmed and ground into powder using a specialized tobacco cyclone mill, then sieved through a 60-mesh screen. After thorough mixing, each sample was divided into three identical portions stored in brown glass bottles with sealing pads. Samples were kept in a −20 °C refrigerator under light-protected conditions until analysis to maximize stability of aromatic compounds. All chemical analyses were completed within 2 weeks of sample preparation.

**TABLE 1:** Information of cigar tobacco leaf samples.

Index	Production areas	Variety/Type	Sample quantity	Harvest year	Processing methods	Aging period (Months)	Key selection criteria
YN-pr	Yunnan Pu’er	‘Yunxue’ no. 1	1	2021	Air-drying	18–24	Typical yunnan light-aroma style, fully matured
YN-mg	Baoshan maguan, yunnan	‘Yunxue’ no. 3	1	2021	Sun-drying	18–24	Rich aroma with pronounced sweetness
HB	Enshi, hubei	‘Eyan’ series	1	2021	Air-drying	12–18	Mellow aroma with ample intensity
NIA	Indonesia (east java)	Besuki NO	1	Import batch	Traditional air-drying	>24	Imported primary ingredients, distinctive flavor profile, serving as international reference
HN	Danzhou, hainan	‘Haixue’ series	1	2021	Air-drying	12–18	Tropical climate characteristics, vivid aroma
SC	Panzhihua, sichuan	‘Chuanyan’ series	1	2021	Air-drying	12–18	Mountainous region characteristics, unique aroma
QL	Yishui, shandong	‘Zhongxue’ no. 1	1	2021	Air-drying	6–12	Representative of northern production areas, serving as geographical and climatic reference

20 mL headspace vials, Shandong Haineng Scientific Instruments Co., Ltd.; DB-5MS quartz capillary column (30 m × 0.25 mm, 0.25 μm), MXT-WAX capillary column (30 m × 0.53 mm, 1.0 μm), Restek Corporation, United States. Gas Chromatography-Olfactometry-Mass Spectrometry (GC-O-MS) system (Agilent 8890 GC + 7000D + Olfactometry Port), extraction head (120 μm DVB/CWR/PDMS), Agilent Technologies, United States. FlavourSpec® Gas Chromatography-Ion Mobility Spectrometry, G.A.S. GmbH (Dortmund, Germany); CTC-PAL 3 Static Headspace Autosampler, CTC Analytics AG (Switzerland, Zwingen); VOCal Data Processing Software (0.4.03), G.A.S. GmbH (Germany, Dortmund).

### 2.2 Test method

#### 2.2.1 SBSE-GC-O-MS

SBSE Analysis Conditions: Weigh 500 mg of sample into a 20 mL headspace vial, then add 20 μL of internal standard solution (saturated NaCl - 10 μg/mL) and 15 mL of hot water at 60 °C. Insert the PDMS magnetic stirring adsorption rotor and seal the headspace vial. Place the headspace vial in a 60 °C constant-temperature water bath magnetic stirrer. After 30 min of adsorption by the rotor, remove the vial. Clean residual material from the stirrer rotor surface with pure water, wipe dry, and immediately transfer to a gas chromatography vial for analysis. Place the gas chromatography vial containing the stirrer rotor into the thermal desorption unit to desorb the adsorbed material from the rotor.

GC-MS Analysis Conditions: DB-5MS quartz capillary column (30 m × 0.25 mm, 0.25 μm); carrier gas: high-purity helium; column flow rate: 1.2 mL/min (constant flow mode); Inlet temperature: 25 °C; Injection mode: Splitless injection; Injection volume: 2 μL; Temperature program: Start at 40 °C, hold for 3.5 min, ramp at 10 °C/min to 100 °C, then at 7 °C/min to 180 °C, finally at 25 °C/min to 280 °C, hold for 5 min. Ion source: EI source; Ionization voltage: 70 eV; Ion source temperature: 230 °C; Transfer line temperature: 280 °C; Scan mode: Full scan; Scan range: 29–400 amu. Qualitative analysis based on total ion current (TIC) profile, retention time, spectral library (NIST17), and retention index. Quantitative analysis using internal standard hexane.

#### 2.2.2 PY-GC-O-MS

Fill the pyrolysis sample tube with approximately 2 mg of quartz wool (pesticide residue grade), compact it (skip this step as the pyrolysis tube is pre-sealed), weigh 1.0 mg of sample (±2%), place it on the quartz wool, then add another 2 mg of quartz wool and compact it. Insert blank control tubes (filled with quartz wool only) between samples to ensure no instrument residue remains. Add 1 μL of pre-prepared n-heptadecane-dichloromethane solution (2.088 μL/mL, with n-heptadecane as internal standard) to each sample. Repeat this procedure three times for each sample.

Specific pyrolysis conditions: Pyrolysis atmosphere consists of a nitrogen-oxygen mixture containing 21% oxygen (air); gas flow rate is 74 mL/min; pyrolysis temperature is 350 °C with a heating duration of 30 s; desorption temperature is 300 °C.

#### 2.2.3 Gas chromatography-mass spectrometry method

HP-5 capillary column (30 m × 0.25 mm × 0.25 μm); carrier gas: helium, flow rate: 1.5 mL/min; split mode with split ratio of 20:1; injector temperature: 300 °C; Temperature program: Hold at 40 °C for 1 min, then ramp at 5 °C/min to 280 °C and hold for 5 min; Solvent delay: 2.5 min; Ionization mode: Electron Impact (EI); Ionization energy: 70 eV; Ion source temperature: 230 °C; Quadrupole temperature: 150 °C; Scan mode: Full scan; Scan range: 29 amu–400 amu.

#### 2.2.4 -Filter pre-treatment method

The determination of particulate matter in flue gas employs central-cutting two-dimensional gas chromatography-mass spectrometry. Particulate matter from four smoke samples is collected on each Cambridge filter. Two parallel filters are placed in a conical flask, with 10 mL of methyl tert-butyl ether solvent and 100 μL of mixed standard solution (4 μg/mL methyl valerate, methyl heptanoate and methyl undecanoate dissolved in methanol) were added. After sealing and shaking for 30 min, the supernatant was transferred to a chromatographic vial for analysis.

One-dimensional column: DB‒5MS column, constant flow rate 1.9 mL/min; Two-dimensional column: DB‒WAX column, constant flow rate 1.9 mL/min; Injector temperature: 250 °C; Injection volume: 3 μL; Injection mode: Splitless injection; Splitless time: 1 min; Purge flow rate: 50 mL/min; Central cutting times: Cut 1 (5.1–10.0 min), Cut 2 (10.0–16.6 min), Cut 3 (16.6–23.5 min), Cut 4 (23.5–30.5 min). One-dimensional temperature program: Initial temperature for all 4 segments is 45 °C (hold for 2 min), then ramp at 6 °C/min. Segment 1 °C–93 °C, Segment 2 °C–132.6 °C, Segment 3 °C–174 °C, Segment 4 °C–216 °C. Rapid cooling to 60 °C (Segments 1 & 2) or 80 °C (Segments 3 & 4). Two-dimensional temperature program: Injection port 1: 4 °C/min to 180 °C, then 10 °C/min to 230 °C (20 min); Injection ports 2 and 3: 4 °C/min to 230 °C (20 min); Injection port 4: 4 °C/min to 230 °C (30 min). GC/MS interface temperature: 240 °C; Electron energy: 70 eV; EI source temperature: 230 °C; Quadrupole temperature: 150 °C; Mass scan range: 33–400 amu; Peak area integration using extracted ion mode.

#### 2.2.5 Sniffing device conditions

MS quadrupole temperature 150 °C, electron impact ion source, transfer line temperature 290 °C, electron energy 70 eV, scan range same as MS conditions. The split ratio between the odor port and MS port was 1:1, with an odor port temperature of 280 °C. For GC-MS/O analysis, five members performed odor descriptions on the same sample to minimize subjectivity, recording odor characteristics and retention times.

### 2.3 Formation and screening of the sniffer panel

GC-O analysis was conducted by a panel of five screened and trained sniffers (3 female, 2 male, aged 25–40). All members are long-term researchers in our laboratory with at least 2 years of experience in food flavor chemistry or sensory science, and are familiar with common odor descriptors. Prior to the formal experiment, the sniffers passed a standard olfactory acuity screening. This included successfully identifying and describing odors from “Olfactory Sensitivity Test Strips” (a series of n-butanol solutions prepared in-house according to the ASTM E679 standard) to ensure normal olfactory function and basic odor discrimination and description capabilities.

#### 2.3.1 Sniffer training

Before the formal analysis, the sniffers underwent targeted training consisting of three sessions (2 h each). The training covered: 1) Principles of the GC-O system and safety operating procedures; 2) Learning a unified odor descriptor vocabulary, established based on prior literature and consensus from preliminary sensory profiling of the overall samples (e.g., “fruity,” “floral,” “green/grassy,” “sweet,” “sour,” etc.); 3) Practice sniffing known standards (such as ethyl acetate, hexanal, linalool, etc.) on the system to become familiar with the airflow sensation at the outlet, the time delay of odor appearance, and the intensity recording method.

#### 2.3.2 GC-O evaluation procedure and data recording

All sniffers analyzed the same sample in individual sniffing booths to avoid mutual interference. The chromatographic conditions were consistent with GC-MS analysis, with the column effluent split in a 1:1 ratio to the mass spectrometer detector and a humidified sniffing port.

Each sniffer was provided with data acquisition software or a recording sheet to record in real-time: the retention time (RT) of perceived odor onset and disappearance, the descriptor for that odor (free description), and the odor intensity. Intensity was assessed using a continuous time-intensity method, where sniffers verbally reported the intensity in real-time on a 0–5 point scale, and the software recorded the intensity curve over time. Ultimately, the maximum perceived intensity (Imax) for each perceivable peak was extracted for subsequent analysis.

#### 2.3.3 Experiment replication and data processing

To ensure result reliability, each sample was evaluated twice by each sniffer. During data processing, only odor-active regions (AR) that were detected in at least three (more than half) sniffers’ repeated evaluations and had broadly consistent descriptors were retained and identified as valid GC-O signals. For each valid AR, its odor description was determined through panel consensus, and its intensity value was taken as the average of the “maximum perceived intensity (Imax)” scores from all sniffers who detected that AR.

### 2.4 Data processing

GC-MS: Qualitative analysis of volatile components primarily relies on mass spectrometry standard libraries and retention index (RI) identification. By searching the NIST standard library (NIST Chemistry WebBook, SRD 69) to identify the CAS number of the substance. The retention index is recorded under the column heading for the polar capillary column used in the experiment. The calculated retention indices (RIs) are compared with the retrieved values; retention is confirmed if the error is less than 50%, indicating qualitative confirmation of the volatile component. RIs for unknown compounds were calculated using retention times obtained for n-alkanes (C7–C30) under identical GC-MS conditions.

Following the methodology described in (Chen D. et al., 2025), odor activity values (OAV) were employed to assess the contribution of each volatile component to the aroma profile of cigar tobacco samples. Odor Activity Value (OAV)calculation: Quantify key compound concentrations via GC-MS, combine with literature-reported odor thresholds, and compute OAV = Concentration/Threshold.

All data were processed using SPSS 27 software. GC-MS data were primarily analyzed using the Maiwei Metabolic Cloud platform, while Origin 2019 software was employed for plotting grid diagrams and correlation heatmaps. Experimental results are presented as mean ± standard error.

## 3 Results and discussion

### 3.1 Analysis of aroma-active components in cigar tobacco leaves (SBSE)

To delineate the inherent aroma profile of cigar tobacco leaves, this study employed stir bar sorption extraction coupled with gas chromatography -Olfactory-Mass Spectrometry (SBSE-GC-O-MS) to systematically analyze the aroma-active components in cigar tobacco leaves. A total of 69 aroma-active compounds were identified. Based on the odor contribution theory, to characterize the flavor profiles of different cigar tobacco leaves and identify key odor compounds revealing stylistic differences, the odor contribution of each volatile compound was calculated using concentration data combined with literature-reported odor threshold values (Table 2). This process identified 13 compounds contributing most significantly to cigar tobacco aroma characteristics. The identification of key aroma compounds was based on a multi-indicator comprehensive screening criterion: compounds must meet relative abundance (≥10%), sensory contribution relative odor activity value ≥1, and intergroup discriminative ability with variable importance projection value ≥ 1, while exhibiting statistical significance. Specifically, they must satisfy one or more of the following conditions. Collectively, these substances form the foundation of the complex and unique intrinsic aroma profile of cigar tobacco leaves.

**TABLE 2:** Key odor-active compounds identified in cigar tobacco leaves (SBSE-GC-O-MS).

Odor description	Compound	RT	RI	Odor threshold (μg/L)	Relative content (%)	OAV	VIP	Possible origin
Woody, citrus (lemon, citrus), fresh pine, floral (rose, saffron, violet), grassy, spicy, herbal/Medicinal, sweet, creamy/Fatty	Cyclohexene, 1-methyl-4-(1-methylethenyl)-, (S)-	10.268	1,031	0.034	0.075	0.899	0.578	Terpenoid biosynthesis
	Bicyclo [3.1.1]heptane, 6,6-dimethyl-2-methylene-, (1S)-	11.996	​	4.16	3.435	0.068	1.025	Terpenoid biosynthesis
	D-limonene	12.905	​	0.034	4.396	0.899	1.182	Terpenoid biosynthesis
	Cyclohexanol, 1-methyl-4-(1-methylethenyl)-, acetate	13.077	1,317	ND	3.356	0	1.117	Terpenoid biosynthesis
	Nonanal	15.44	1,104	0.001	0.057	604.732	0.143	Fatty acid oxidation
	Megastigmatrienone	31.508	1,473	ND	1.306	0	0.759	Carotenoid degradation
	3,7,11,15-Tetramethyl-2-hexadecen-1-ol	39.35	2,116	ND	8.022	0	3.152	Chlorophyll/Phytol degradation
	Neophytadiene	38.237	1837	ND	16.910	0	3.799	Chlorophyll/Phytol degradation
Amine-like/Aminic	6-Quinolinamine, 2-methyl-	28.349	​	ND	1.682	0	1.512	Maillard reaction
Smoky	Pyridine, 3-(1-methyl-2-pyrrolidinyl)-, (S)-	24.39	1,361	470	42.828	1.086	7.684	Pyrolysis products of alkaloids
Odorless/No distinct odor	1,5,9,11-Tridecatetraene, 12-methyl-, (E, E)-	13.781	​	ND	1.247	0	1.176	Carotenoid degradation
	Pyridine, 3-(3,4-dihydro-2h-pyrrol-5-yl)-	26.747	1,427	ND	1.382	0	0.620	Pyrolysis products of alkaloids
	2,3′-dipyridyl	30.009	1,556	ND	6.058	0	1.948	Pyrolysis products of alkaloids

Based on the results in Table 2, the intrinsic aroma profile of cigar tobacco leaves centers on woody, fruity (citrus, lemon), fresh pine, floral (rose, saffron, violet), grassy, spicy, herbal, sweet, and resinous notes, underpinned by woody and smoky undertones contributed by lignin degradation products. Analysis reveals that D-limonene and (S)-β-pinene impart citrus and subtly spicy freshness (Chen, 2011), (+)-α-Pinene and neopentadiene establish the foundation of pine, resin, and green notes. The harmonious balance between neopentadiene and carotenoid degradation components likely explains tobacco’s distinctive fresh-aromatic profile and mellow aroma (Yang et al., 2005; Zhou et al., 2005). Nonanal contributes rose-like floral notes at low concentrations, intertwining with fruity aromas to enhance the fragrance’s opulence and sweetness (Zhu et al., 2016). Terpineol acetate acts as a “bridge” with its fresh herbal and subtle floral notes, facilitating a more natural transition between fruity, floral, and woody aromas, resulting in a softer and fuller overall fragrance. Triterpenes provide deep fruity-woody-tobacco notes (Baker et al., 2004; Jiang, 2025; Rodgman et al., 2013). These compounds primarily originate from the degradation of carotenoids and the breakdown and transformation of terpenoids (Skopp et al., 2017; Yang et al., 2020).

### 3.2 Analysis of aromatic active components in pyrolytic products of cigar tobacco leaves

To elucidate the aroma profile of cigar tobacco leaves during pyrolysis, pyrolysis coupled with gas chromatography -Olfactory-Mass Spectrometry (PY-GC-O-MS) to systematically analyze the aroma-active components in cigar tobacco leaves. A total of 63 aroma-active compounds were identified. Based on odor contribution theory, to characterize the flavor profiles and key odor compounds of different cigar tobacco leaves under pyrolysis conditions, the odor contribution of each volatile compound was calculated using concentration data combined with literature-reported odor threshold values (Table 3). This process identified 27 compounds contributing most significantly to the aromatic characteristics of cigar tobacco leaves. The identification of key aroma compounds was based on a multi-indicator comprehensive screening criterion: compounds must meet relative abundance (≥10%), sensory contribution relative odor activity value ≥1, and intergroup discriminative ability with variable importance projection value ≥1, while exhibiting statistical significance. Specifically, they must satisfy one or more of the following conditions. Collectively, these substances form the foundation of the complex and unique intrinsic aroma profile of cigar tobacco leaves.

**TABLE 3:** Key odor-active compounds identified in cigar tobacco leaves (PY-GC-O-MS).

Odor description	Compound	RT	RI	Odor threshold (μg/L)	Relative content (%)	OAV	VIP	Possible origin
Sweet, caramel, buttery, toasted/Nutty (almond-like), and toasty/Bready, with a slight sourness/Tang	2-Propanone, 1-hydroxy-	4.492	665	ND	3.499	0	1.185	Maillard reaction
	2-Propanone, 1-(acetyloxy)-	8.658	870	ND	0.989	0	1.332	Maillard reaction
	1,2-Cyclopentanedione, 3-methyl-	13.683	1,043	0.026	0.502	10.318	0.523	Maillard reaction
	Furaneol	15.074	1,070	0.0015	0.128	41.517	0.373	Maillard reaction
Floral, black tea, herbal, fruity	Indole	21.239	1,295	0.04	0.685	9.566	0.744	Shikimate pathway
	7-Oxabicyclo [4.1.0]heptan-3-ol, 6-(3-hydroxy-1-butenyl)-1,5,5-trimethyl-	29.854	1,674	ND	1.902	0	1.003	Chlorophyll degradation
	Neophytadiene	33.243	1837	ND	1.80	0	0.797	Chlorophyll degradation
	Phytol	38.377	2,114	0.64	0.823	0.705	1.228	Chlorophyll degradation
Sour, medicinal, amine-like odor	Acetic acid	4.032	610	0.013	2.653	114.36	0.62	Maillard reaction
	Phenol	12.707	980	0.021	2.113	54.987	0.923	Lignin degradation
	Phenol, 2-methoxy-	15.183	1,090	0.0015	0.174	61.97	0.555	Lignin degradation
	Pyrrolidine, 1-(1-pentenyl)-	19.423	​	ND	0.530	0	1.072	Maillard reaction
Typical fatty, cucumber peel, and waxy notes	2-Nonen-1-ol, (E)-	15.551	1,176	0.209	1.120	2.985	1.123	Lipoxygenase pathway
Smoky/Unknown flavor	4-Pyridinol	17.272	1,154	ND	2.448	0	1.417	Hydroxylated pyridine derivatives
	3-Pyridinol	17.433	​	ND	1.937	0	1.358	Hydroxylated pyridine derivatives
	Catechol	19.176	1,205	8	1.217	0.076	1.853	Pyrolysis products of lignin
	Pyridine, 2-(1-methyl-2-pyrrolidinyl)-	21.92	1,354	ND	22.07	​	3.091	Tobacco alkaloids and their metabolites
	Pyridine, 3-(1-methyl-2-pyrrolidinyl)-, (S)-	22.523	1,361	470	30.659	0.035	3.257	Tobacco alkaloids and their metabolites
	Pyridine, 3-(3,4-dihydro-2h-pyrrol-5-yl)-	24.309	1,427	ND	3.274	0	2.128	Tobacco alkaloids and their metabolites
	2H-1,2-oxazine, tetrahydro-2-methyl-6-(3-pyridinyl)-, (−)-	25.523	​	ND	0.344	0	1.007	Tobacco alkaloids and their metabolites
	1,2,3,6-Tetrahydro-2,3′-bipyridine	26.52	​	ND	0.275	0	1.120	Tobacco alkaloids and their metabolites
	2,3′-dipyridyl	26.858	1,556	ND	2.133	0	1.129	Tobacco alkaloids and their metabolites
	Cotinine	30.824	1721	ND	1.211	0	1.284	Tobacco alkaloids and their metabolites
	n-Hexadecanoic acid	35.624	1968	ND	1.078	0	1.18	Fatty acids
​	Thunbergol	37.852	2073	ND	0.386	0	1.002	Fatty acids
	4,8,13-Cyclotetradecatriene-1,3-diol, 1,5,9-trimethyl-12-(1-methylethyl)-	37.878	​	ND	0.447	0	1.009	Degradation products of cembranoids
	Octadecanoic acid	39.261	2,172	ND	0.291	0	0.694	Degradation products of cembranoids

Based on the results in Table 3, the aroma profile of cigar tobacco during pyrolysis centers on sweet, caramelized sweet, fruity, and fresh herbaceous notes, underpinned by tobacco-like and earthy aromas derived from pyridine and pyrrole nitrogen-containing heterocyclic compounds. Analysis reveals that 1,2-Cyclopentanedione, 3-methyl-(3-methyl-1,2-cyclopentanedione) contributes the characteristic caramelized roasted aroma and malty sweetness, forming the baked notes within the base profile. 2-Propanone, 1-(acetyloxy)- (acetoxyacetone) provides subtle notes of fruity acidity and fatty aromas. Blending with caramel, it adds dimensionality to the complex sweetness. Furaneol, a representative furanone, imparts pronounced fruity sweetness and caramel-nutty notes, serving as a key component enhancing aroma distinctiveness (An et al., 2025). Indole exhibits soft floral and animalic notes at low concentrations, adding a mysterious and weighty undertone to the overall aroma. 7-Oxabicyclo [4.1.0]heptan-3-ol, 6-(3-hydroxy-1-butenyl)-1,5,5-trimethyl- (an epoxy monoterpenol) releases delicate herbal freshness and subtle woody notes during pyrolysis, sustaining the aroma’s natural character and crispness (Ma et al., 2020; Zhu et al., 2016). Neophytadiene and Phytol jointly form the foundation of tobacco leaf’s green and mildly oily notes. The transformation of neophytadiene particularly enhances the aroma’s moistness and astringency. SBSE-GC-O-MS comparisons reveal that the “baked sweetness,” “caramel-nutty notes,” and ”fresh grassy texture” in cigar tobacco during high-temperature pyrolysis are not the direct volatilization of original substances. Instead, they result from Maillard and caramelization reactions between polysaccharides and amino acids at high temperatures, producing pyranones, furanones, and other compounds. Simultaneously, the pyrolysis and transformation of monoterpenes like α-pinene and neopentadiene form the woody forest and fresh herbal notes in the aroma’s finish. The generation and retention of heterocyclic compounds such as indole impart a complex, deep floral-earthy base to the overall aroma beyond its rich sweetness. This series of transformations demonstrates that the signature aroma profile of cigar tobacco is the result of synergistic interactions and conversions among precursor substances—including sugars, amino acids, lipids, and terpenes—under high temperatures. Together, these elements construct its multi-layered, multidimensional flavor characteristics (Huang et al., 2022; Mottram, 2007).

### 3.3 Analysis of aromatic active components in cigar smoke

To characterize the aroma profile of cigar tobacco leaves during pyrolysis into smoke, a gas chromatography-gas chromatography-optical-mass spectrometry (GC-GC-O-MS) system was employed. -Olfactory-Mass Spectrometry (GC-GC-O-MS) to systematically analyze the aroma-active components in cigar tobacco leaves. A total of 69 aroma-active compounds were identified. Based on odor contribution theory, to characterize the flavor profiles and key odor compounds of different cigar tobacco leaves under pyrolysis conditions, the odor contribution of each volatile compound was calculated using concentration data combined with literature-reported odor threshold values (Table 4). This process identified 20 compounds contributing most significantly to the aromatic characteristics of cigar tobacco leaves. The identification of key aroma compounds was based on a multi-indicator comprehensive screening criterion: compounds must meet relative abundance (≥10%), sensory contribution relative odor activity value ≥1, and intergroup discriminative ability with variable importance projection value ≥1, while exhibiting statistical significance. Specifically, they must satisfy one or more of the following conditions. Collectively, these substances form the foundation of the complex and unique intrinsic aroma profile of cigar tobacco leaves.

**TABLE 4:** Key odor-active compounds identified in cigar tobacco leaves (Smokiness).

Odor description	Compound	RT	RI	Odor threshold (μg/L)	Relative content (%)	OAV	VIP	Possible origin
Fruity, wine-like, sweet, with a slight waxy note	Formic acid, butyl ester	13.546	724	0.37	0.093	3.113	0.121	Esterification
	Butanoic acid, 2-methyl-	68.061	861	0.02	0.025	15.229	0.25	Esterification
	1,2-Ethanediol, diacetate	55.047	994	1.8	0.914	6.285	1.316	Esterification
Nutty, caramel-like	Pyrazine, methyl-	41.69	831	1.9	0.158	1.028	0.188	Maillard reaction
	Pyrazine, 2,6-dimethyl-	45.829	917	ND	0.143	0	2.397	Maillard reaction
	2-Propanone, 1-hydroxy-	46.263	917	1.72	0.119	1.031	0.141	Maillard reaction
	Pyrazine, 2-ethyl-5-methyl-	50.715	1,005	0.036	0.022	7.513	0.182	Maillard reaction
Fruity, green/grassy, woody	Phenylethyl alcohol	81.603	1,116	0.012	0.086	88.38	0.16	Chlorophyll degradation
	Neophytadiene	81.878	1837	ND	1.246	0	2.015	Chlorophyll degradation
Amine odor, sour odor, unpleasant odor	Acetic acid	54.38	610	0.013	1.005	957.47	2.082	Short-chain fatty acids
	Butanoic acid, 3-methyl-	68.061	863	0.0018	0.047	323.69	0.132	Short-chain fatty acids
	Retinol, acetate	100.593	2,555	ND	2.613	0	1.893	Carotenoid degradation
Smoky/Unknown flavor	Hydroxylamine	16.152	​	ND	1.629	0	2.35	Tobacco alkaloids and their metabolites
	Glycidol	55.235	755	ND	0.649	0	1.757	Epoxide hydrolysis
	Propylene glycol	64.604	740	16	5.963	4.614	4.639	​
	Pyridine, 3-(1-methyl-2-pyrrolidinyl)-, (S)-	79.68	1,361	16	1.42	0	0.214	Diol oxidation
	Phenol	86.074	980	0.00017	44.72	1.178	3.086	Amide bond hydrolysis
	Triacetin	89.373	1,344	ND	25.42	0	4.765	Tobacco alkaloids and their metabolites
	Pyridine, 3-(3,4-dihydro-2h-pyrrol-5-yl)-	96.156	1,427	ND	2.005	0	1.510	Esterification
	1,2,3-Propanetriol, 1-acetate	96.725	1,093	ND	0.183	13,336.36	0.255	Esterification

Based on the results in Table 4, the final aroma profile of cigar smoke centers on roasted, nutty, sweet, floral, woody, and buttery notes, underpinned by tobacco-like, earthy, and satisfying undertones derived from pyrazine and pyridine nitrogen-containing heterocyclic compounds. Analysis reveals that the “pyrazine triad”—comprising methyl-, 2,6-dimethyl-, and 2-ethyl-5-methyl-pyrazine—consistently delivers roasted almond and toasted bread aromas, establishing the dominant charred nutty character (Erten and Cadwallader, 2017; Yan et al., 2021). Ethanone, 1-(1H-pyrrol-2-yl)-1-(1H-pyrrol-2-yl)ethanone, serves as a “bridge” with its distinctive smoky earthy aroma, tightly linking nutty notes with tobacco undertones to impart greater richness and depth to the overall fragrance (Chi et al., 2019). Formic acid, butyl ester delivers a refreshing fruity-sweet top note, effectively enhancing the pleasantness and complexity of the smoke; while Phenylethyl Alcohol gently introduces a low-threshold rose-honey floral note, softening the dry intensity of pyridine and bridging to the subtle waxy-violet powder aroma of Retinol acetate. This creates a velvety “floral-resinous” coating on the smoke, delivering a denser nasal sensation. As the soul component of the cigar, Pyridine, 3-(1-methyl-2-pyrrolidinyl)-, (S)- (nicotine) not only delivers the signature tobacco character and strength, but its pyrolysis derivatives are also a crucial source of the smoke’s complex earthy notes. In summary, the evolution of this round’s smoke aroma has transcended a “single-path accumulation” model, entering an “interactive synergy” phase: elements like “baked sweetness,” “caramelized nutty notes,” and “fresh grassy texture” emerge from the complex interactions and synergistic effects of multiple chemical compounds undergoing intricate reactions—including the Maillard reaction, caramelization, and monoterpene pyrolysis transformations (Chen Z. et al., 2025). These key aromatic compounds—such as 3-methyl-1,2-cyclopentanedione, acetoxyacetone, furfural, indole, epoxy monoterpenols, neopentadiene, and phytol, each contribute distinctive aromas while collectively constructing the complex and balanced flavor profile of cigar smoke through an “interaction enhancement” mechanism. This delivers a unique and rich sensory experience to the smoker (Jiang et al., 2024; Mitsui et al., 2015).

### 3.4 Quantitative evolution analysis of key aroma compounds in the “tobacco leaf-pyrolysis-tobacco smoke” pathway

**FIGURE 1:**
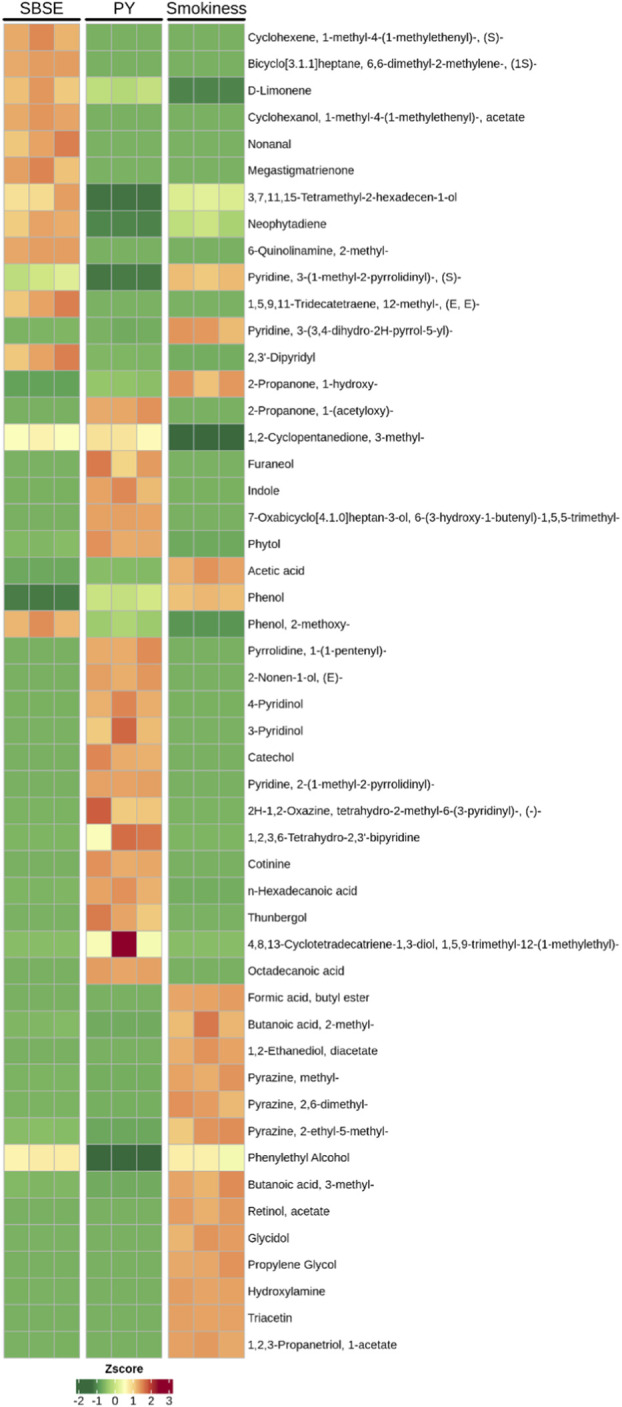
Quantitative evolution analysis of representative key aroma compounds.

To quantitatively elucidate the dynamic evolution of cigar aroma from tobacco leaves to smoke, key representative compounds were selected for systematic comparison of their relative abundances across three sample categories: SBSE (raw tobacco leaves), PY (pyrolysis products), and smoke (Smokiness). As shown in Figure 1, the analysis revealed that D-Limonene (detected at zero concentration in Smokiness), Phenol, and certain pyridine compounds were present in all three categories (all concentrations non-zero). SBSE-specific compounds included Cyclohexene, isocycloheptane derivatives, 5,9,11-Tridecatetraene, 2,3′-Dipyridyl, and Megastigmatrienone. PY-specific compounds included Furaneol, Indole, Catechol, 4-Pyridinol, 3-Pyridinol, and the pyridine derivative Cotinine. Smokiness-specific compounds: formic acid butyl ester, Triacetin (extremely high concentration, approx. 325), Propylene Glycol (approx. 80–90), Retinol acetate (approx. 45–47), various esters, alcohols (e.g., Glycidol, Hydroxylamine).

By calculating relative intensity ratios and generation/loss factors, we aimed to quantify direct transfer efficiency, thermal generation intensity, and precursor conversion contributions. Compounds eligible for DTE calculation (13 in total) are listed in Table 5. High concentrations of pyridine, 3-(3,4-dihydro-2H-pyrrol-5-yl)-, acetic acid, 2-Propanone, 1-hydroxy- are not primarily transferred directly from tobacco leaves. Instead, they are generated in large quantities during pyrolysis (PY) and especially combustion (Smokiness) stages through intense thermochemical reactions (e.g., Maillard reaction, Strecker degradation, polymer pyrolysis). Hydroxypropanone, acetic acid, and short-chain esters/alcohols exhibit near-zero concentrations in SBSE. Although PY concentrations are low, they significantly enrich in Smokiness (reaching 14.3), indicating efficient vaporization upon formation. Pyrazine, methyl-, and Pyrazine, 2,6-dimethyl- exhibit DTE values as high as 40–50, indicating “pyrolysis” as the key chemical switch for baking-nutty aromas. Characteristic tobacco compounds like Pyridine, 3-(1-methyl-2-pyrrolidinyl)-, and (S)-alkaloid-nicotine undergo highly efficient transfer (TGF≈0.06, DTE≈30), ensures peak physiological intensity and characteristic tobacco flavor at the inhalation end. Neophytadiene, despite its extremely high content in tobacco leaves (>35), only approximately 44% enters the smoke; it exhibits a typical “partial loss” profile, likely due to retention in the tar phase caused by its high molecular weight and low vapor pressure. Phenol exhibits a DTE far exceeding 1, indicating not only efficient release of pre-existing phenol but also substantial new formation; its TGF > 8 demonstrates that pyrolysis strongly promotes its generation.

**TABLE 5:** Relative intensity ratio (RIR), direct transfer efficiency (DTE), and precursor contribution index (PCI) quantitative analysis.

Index	Avg (SBSE)	Avg (PY)	Avg (Smokiness)	DTE (Smk/SBSE)	RIR (Smk/SBSE)	RIR (Smk/PY)	PCI (PY/Smk)	Notes
1,2,3-Propanetriol, 1-acetate	0.0709	0	26.54	374.3	374.3	—	0	High-efficiency release, no thermogenesis
2-Propanone, 1-hydroxy-	0	2.36	14.32	—	—	6.07	0.165	Complete thermogenesis, partial entry into flue gas
3,7,11,15-Tetramethyl-2-hexadecen-1-ol	0.071	0	0.0438	0.617	0.617	—	0	Present in leaves but limited release
Acetic acid	0.0033	1.22	12.86	3,897	3,897	10.54	0.095	Extremely enriched in flue gas, primarily thermogenic
Butanoic acid, 2-methyl-	0.014	0	0.2209	15.78	15.78	—	0	Primordial release type
Butanoic acid, 3-methyl-	0.0319	0	0.5362	16.8	16.8	—	0	Primordial release type
Glycidol	0.0466	0	13.17	282.6	282.6	—	0	High-efficiency release, non-thermogenic
Hydroxylamine	0.0493	0	16.93	343.4	343.4	—	0	High-efficiency release, non-thermogenic
Neophytadiene	35.12	1.26	15.44	0.439	0.439	12.25	0.0816	High *in-situ*, low transfer, weak thermogenesis
Phenol	0.1435	1.1812	2.221	15.48	15.48	1.88	0.532	Significant thermogenesis + high-efficiency release
Pyrazine, methyl-	0.0481	0	1.939	40.31	40.31	—	0	High release efficiency, thermal generation
Pyrazine, 2,6-dimethyl-	0.0466	0	2.463	52.85	52.85	—	0	High release efficiency, thermal generation
Pyrazine, 2-ethyl-5-methyl-	0.0305	0	0.2839	9.31	9.31	—	0	High release efficiency, thermal generation
Pyridine, 3-(1-methyl-2-pyrrolidinyl)-, (S)-	355.15	20.87	653.89	1.84	1.84	31.33	0.0319	Primarily from primary release, minor thermal generation
Pyridine, 3-(3,4-dihydro-2h-pyrrol-5-yl)-	3.032	1.7698	42.97	14.17	14.17	24.28	0.0412	High release efficiency + moderate thermal generation

The Relative Intensity Ratio (RIR) measures the distribution preference of a component across different samples: if the SBSE, value is 0, only the presence or absence of PY, formation is considered; the opposite applies. Direct Transfer Efficiency (DTE) evaluates the ability of existing components in tobacco leaves to transfer into smoke: If DTE ≈1: essentially equal transfer; If DTE >> 1: potential concentration effect or co-distillation-enhanced release; If DTE << 1: prone to degradation or retention in residues. Thermal Generation Factor (TGF) evaluates whether the compound is primarily newly generated during pyrolysis: If TGF >> 1: Significant thermal generation; If SBSE, 0 and PY > 0: Fully thermally generated. Precursor Contribution Index (PCI) identifies certain “indirect products” that show no signal in SBSE, or PY, but appear in Smokiness.

### 3.5 Correlation analysis and formation mechanism of key aroma compounds

**FIGURE 2:**
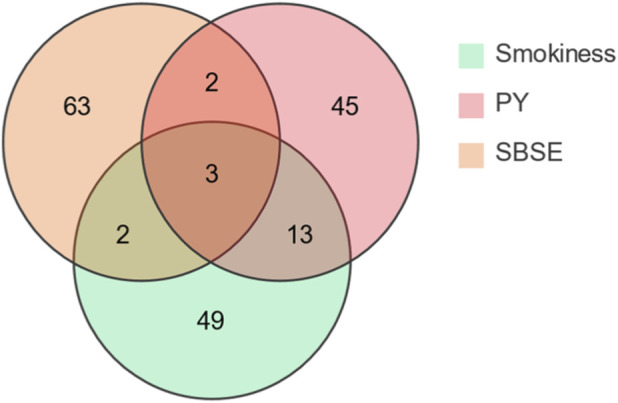
Venn diagram illustrating the overlap of key odor-active compounds among cigar tobacco leaf (SBSE), pyrolysis products (PY), and mainstream smoke.

This study reveals that the aroma profile of cigars undergoes a dynamic and highly selective evolution from tobacco leaf to smoke. As shown in Figure 2, 63 aroma compounds exist exclusively in the tobacco leaf itself, while only 45 aroma compounds remain after pyrolysis. Five compounds are shared between leaf tobacco and smoke (likely transferred directly). Five compounds are shared between leaf tobacco and pyrolysis (likely thermally generated), while three compounds are common to all three stages.

**FIGURE 3:**
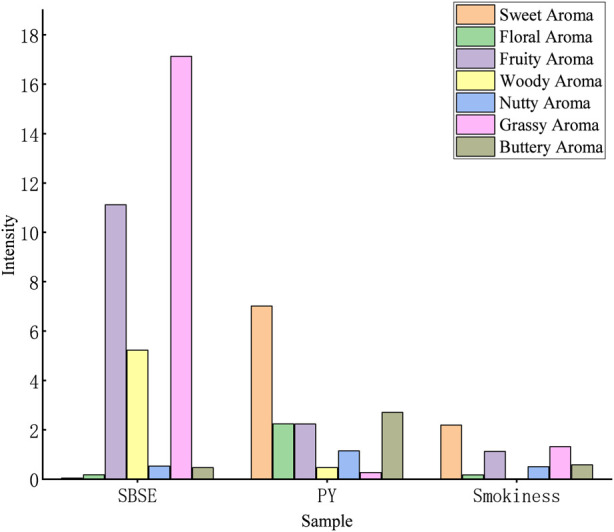
Histogram of aroma profiles of tobacco leaf, pyrolysis products, and smokiness based on sensory attributes.

As shown in Figure 3, sensory profiling analysis of both raw cigar tobacco leaves and pyrolyzed samples revealed significant differences in their aroma profiles. The raw cigar tobacco samples exhibited balanced and rich aroma characteristics, with the highest intensity in green and fruity notes. Pyrolyzed samples were dominated by sweet and caramelized sweet aromas. Notably, the smoke aroma was also dominated by sweetness, while green and fruity notes positively influenced the overall flavor harmony.

**FIGURE 4:**
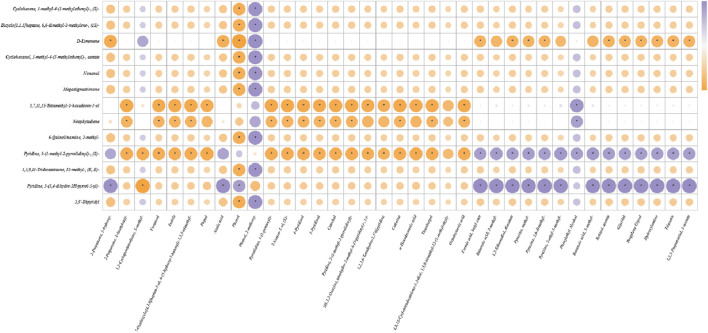
Correlation analysis of key aroma compounds along the “tobacco leaf - pyrolysis - smoke” pathway.

To thoroughly investigate the intrinsic relationship among the inherent aroma of cigar tobacco leaves, pyrolysis products, and the final smoke aroma, this study conducted Pearson correlation analysis on key aroma compounds across three datasets and constructed a correlation network diagram to visualize significant strong correlations (|r| > 0.8, p < 0.01) (Masuda et al., 2025; Pardo-Diaz et al., 2022; Toubiana et al., 2016; Toubiana and Maruenda, 2021). As shown in Figure 4, pyridine and 3-(3,4-dihydro-2H-pyrrol-5-yl)- in the tobacco leaf itself exhibit extremely significant positive correlations with the vast majority of key aroma components in the smoke (except for phenylethyl alcohol). Notably, nicotine in the tobacco leaf itself exhibits a highly significant positive correlation with approximately 80% of the components in the smoke, while showing a highly significant negative correlation with approximately 80% of the components in the pyrolysis products. This phenomenon reveals that nicotine is released in large quantities at high temperatures and transferred to the smoke, and its pyrolysis behavior may inhibit the formation or accumulation of certain thermal reaction products. Phenol, 2-methoxy- (2-methoxyphenol) in pyrolysis products showed significant correlations with multiple terpenes and their derivatives in the tobacco leaf itself (e.g., Cyclohexene, 1-methyl-4-(1-methylethenyl)-, (S)-, D-limonene, cyclohexanol, 1-methyl-4-(1-methylethenyl)-, acetate), aldehydes (nonanal), carotenoid degradation products (megastigmatrienone), and nitrogen-containing heterocycles (6-quinolinamine, 2-methyl-, 2,3′-dipyridyl) all exhibit extremely significant positive correlations. Conversely, phenol itself shows extremely significant negative correlations with these substances. Additionally, 3,7,11,15-Tetramethyl-2-hexadecen-1-ol (Phytol) and Neophytadiene present in the tobacco leaf itself showed significant negative correlations with multiple pyrolysis products (e.g., 2-Propanone, 1-(acetyloxy)-, furaneol, phytol, phenol, and its methoxy derivatives) in pyrolysis products, further supporting the pathway where phytol and its derivatives, as important precursors, are consumed and converted into other aroma compounds during pyrolysis. Crucially, D-Limonene present in the tobacco leaf itself exhibited a highly significant negative correlation with a series of substances in the smoke, including esters, pyrazines, alcohols, and glycerol ester derivatives. This directly confirms the instability of this monoterpene during combustion. Its extensive decomposition likely results in the loss of its own aromatic characteristics and may contribute to or promote the formation of other smoke components, particularly those generated through esterification, Maillard reactions, and other pathways.

**FIGURE 5:**
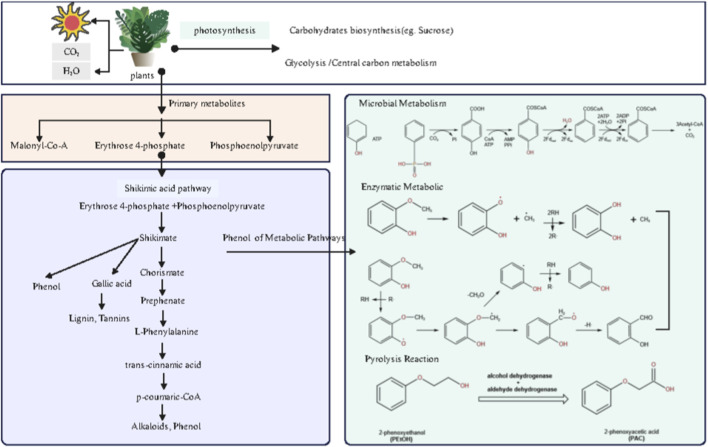
Proposed mechanistic pathways for key aroma compound formation.

As shown in Figure 4 and the corresponding data analysis, 2-methoxyphenol exhibits a highly significant positive correlation with over ten chemical components in tobacco leaves (including terpenoids, aldehydes, carotenoid degradation products, and nitrogen-containing heterocyclic compounds). In contrast, its structural analog phenol shows a highly significant negative correlation with the same set of tobacco leaf components. This stark contrast between positive and negative correlations strongly suggests that during pyrolysis, despite both being phenolic compounds, the formation of 2-methoxyphenol and phenol may be differentially regulated by distinct precursor combinations within the tobacco matrix, or may involve some competitive reaction mechanism. 2-Methoxyphenol typically exhibits smoky, woody, or sweet aromatic characteristics and is a significant contributor to tobacco flavor. Its predicted metabolic pathway is shown in Figure 5. As a key intermediate in biomass combustion, 2-methoxyphenol typically forms secondary organic aerosol (SOA) alongside phenol and 2,6-dimethoxyphenol (Yee et al., 2013). This indicates biomass (e.g., lignin) as its primary biogeochemical precursor source. Lignin pyrolysis products contain not only 2-methoxyphenol but also other methoxyphenolic compounds (Meng et al., 2020). Furthermore, the primary pathway for synthesizing phenolic compounds in plants is the shikimic acid pathway. This pathway begins with erythrulose-4-phosphate and phosphoenolpyruvate, producing shikimic acid, which is then converted into secondary metabolites like flavonoids and phenolics (Borah et al., 2024). These primary metabolites can undergo extensive conversion into phenolic compounds under UV-B radiation, indicating that environmental factors also influence the generation and transformation of phenolic precursors (Liu et al., 2020). The metabolic pathways of 2-methoxyphenol are complex and diverse, involving microbial degradation, enzymatic conversion, and pyrolytic transformation. Anaerobic bacteria exhibit unique enzymatic processes in phenolic compound degradation, such as ATP-dependent phenol carboxylation, which converts phenol to 4-hydroxybenzoic acid via a phenylphosphate intermediate (Boll and Fuchs, 2005). 2-Methoxyphenol can be transformed by multiple enzymes. For instance, the cytochrome P450 CYP102A1 (P450 Bm3) variant oxidizes multiple di-substituted benzenes, including 2-methoxytoluene, yielding two aromatic oxidation products (Munday et al., 2017). This suggests P450 enzyme systems may play a role in metabolizing 2-methoxyphenol derivatives. *In vivo* metabolism and pharmacokinetic studies have identified phenoxyacetic acid (PAA) as the primary metabolite of 2-phenoxyethanol, further demonstrating the critical role of alcohol dehydrogenase and aldehyde dehydrogenase in the oxidation of aromatic alcohols (Hewitt et al., 2022; Kochs et al., 2023). This oxidative dehydrogenation mechanism may also act on the methoxy group in 2-methoxyphenol. Under elevated temperatures, 2-methoxyphenol undergoes pyrolysis. Studies indicate two pathways for its thermal decomposition: the methoxyl cleavage pathway (methoxyl O–C bond cleavage yielding methane and 1,2-dihydroxybenzene) and the induction pathway (phenolic hydroxyl hydrogen abstraction by a cinnamyl radical, followed by intramolecular hydrogen transfer to produce phenol, 2-hydroxybenzaldehyde, etc.) (Dorrestijn and Mulder, 1999).

## 4 Conclusion

This study comprehensively elucidates the dynamic evolution of cigar aromas from tobacco leaves to smoke by systematically integrating GC-O-MS data from Stirred Barrel Sorption Extraction (SBSE), Pyrolysis (Py), and mainstream smoke, combined with Odor Contribution (ROAV) and statistical analysis. Key findings are as follows: The inherent aroma of cigar tobacco leaves is dominated by terpenes and carotenoid degradation products, presenting a profile characterized by “green notes and citrus fruitiness.” Following pyrolysis, the aroma profile shifts to a “toasty sweetness and roasted nutty aroma” centered on furan and pyrazine compounds. The final smoke aroma presents as sweet, green, and fruity. Comparing relative abundances across SBSE, PY, and Smokiness samples reveals that D-Limonene, Phenol, and certain pyridine compounds persist throughout the leaf-to-smoke pathway; SBSE enriched cycloalkenes and diterpenes, PY enriched furanones, indoles, and catechols, while smoke predominantly contained short-chain polar compounds like triacetin, propylene glycol, hydroxyacetone, and acetic acid. Direct transfer efficiency (DTE) for 13 quantifiable compounds revealed: Nicotine DTE ≈30, ensuring physiological potency; Pyrazine DTE 40–50, acting as the “cracking switch” for roasted-nutty aroma; Phenol DTE > 1 with TGF > 8, revealing substantial thermal synthesis; Neophytadiene, despite tobacco leaf abundance >35%, only 44% enters smoke, classified as “partially lost.” Correlation network analysis indicates significant association between furfural (furfuryl) roasting aroma and carotenoid degradation products (neophytadiene) in leaf material. Nutty aroma (2,6-dimethylpyrazine) in smoke shows strong positive correlation with α-terpineyl acetate in leaf material. The final aroma profiles of pyrolysis and smoke are determined by a complex network involving three pathways: direct transfer of inherent tobacco aromas, pyrolysis-generated compounds, and precursor transformations.

## Data Availability

The original contributions presented in the study are included in the article/supplementary material, further inquiries can be directed to the corresponding author.
